# Satellite Remote Sensing Reveals More Beneficial Effect of Forest Plant Diversity on Drought Resistance in More Arid Areas of Yunnan, China

**DOI:** 10.1002/ece3.70999

**Published:** 2025-02-18

**Authors:** Guotao Ma, Hao Sun, Keke Hu, Hong Zhou

**Affiliations:** ^1^ College of Geoscience and Surveying Engineering China University of Mining and Technology‐Beijing Beijing China; ^2^ Inner Mongolia Research Institute China University of Mining and Technology‐Beijing Ordos China; ^3^ Research Center of Applied Geology of China Geological Survey Chengdu Sichuan China

**Keywords:** beneficial effect, drought resistance, forest plant diversity, forest responses to drought stress, satellite remote sensing, Sentinel‐2

## Abstract

Plant diversity is important in enhancing an ecosystem's drought resistance. However, the relationship between plant diversity and drought resistance has historically aroused much controversy. Given that most previous studies on the relationship were conducted with in situ data at a small or point scale, this study explored the relationship with satellite remote sensing, taking Yunnan Province of China as the study area. Specifically, Sentinel‐2 remote sensing data were used to estimate plant diversity. The temporal correlation between the standardized vegetation index (SVI) and standardized precipitation evapotranspiration index (SPEI) was used to express the vegetation sensitivity to drought or drought resistance. A moving window method was developed to explore the relationship between plant diversity and drought resistance. MODIS and SPEI data from 2000 to 2018, as well as Meteorological reanalysis data from 1990 to 2020, were utilized. Results indicated that (1) the remotely sensed plant diversity index was found significantly correlated with field investigations of plant diversity in the study area, with a correlation coefficient of around 0.43–0.49 and *p*‐value < 0.001. The plant diversity of the study area can be recognized with the Sentinel‐2 remote sensing data. (2) The area proportion of having positive temporal correlation coefficients between SVIs and one‐month SPEI varied around 60%–88% during the dry seasons, while that varied around 30%–50% during the wet seasons. Most of the forest vegetation in the study area was sensitive to drought in the dry seasons rather than in the wet seasons. (3) About 80% of the study area presented a beneficial effect of plant diversity on drought resistance, that is, the higher the plant diversity, the lower the forest sensitivity to drought. (4) The beneficial effect of plant diversity has different manifestations in different regions, and it was stronger in more arid and drought‐prone areas.

## Graphical abstract

Forest diversity can be recognized using Sentinel‐2 remote sensing data. Forest was more sensitive to drought in the dry seasons and less sensitive in the wet seasons. Forest diversity presents a beneficial effect on enhancing drought resistance. The beneficial effect was more pronounced in more arid and drought‐prone areas.

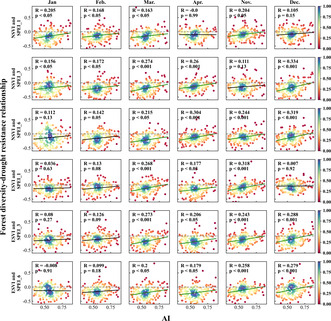

## 1 Introduction

On the fifteenth session of the Conference of the Parties (COP15) of the United Nations Convention to Combat Desertification (UNCCD) in Abidjan, Côte d'Ivoire, the UNCCD Secretariat released ‘Drought in Numbers 2022’. The facts and figures of this report point in the same direction: the duration and severity of droughts are becoming more and more severe, affecting not only human societies but also the ecosystems on which all life depends including our species (Sun et al. 2023b; Tsegai et al. 2022). It was argued that we are standing at a crossroads and we need to find solutions rather than continue destructive actions. Therefore, how to consolidate and enhance the drought resistance of land surface ecosystems is a very valuable and meaningful issue, where drought resistance of a vegetation ecosystem can be simply understood as the capacity to maintain vegetation growth state under the threat or disturbance of droughts (Liu et al. 2022; Van den Berge et al. 2014). As we know, the establishment and maintenance of plant diversity is one of the few available management options for solving this issue (Liu et al. 2022).

Plant diversity can be defined as a combined measure of the number of plant species and the number of plant individuals in each species for a vegetation ecosystem. It has long been acknowledged as crucial for regulating ecosystems' functioning, including lowering their sensitivity to pressures caused by the environment (Grossiord 2020). Many studies have explored whether plant diversity can alleviate the adverse effects of drought on ecosystems. To our best knowledge, there are at least three main recognitions: (1) Plant diversity plays a positive role in mitigating the impact of drought on vegetation, which can be called the benefit effect of plant diversity on drought resistance of vegetation (abbreviated as diversity benefit) (Isbell et al. 2015; Lebourgeois et al. 2013; Pretzsch et al. 2013; Steckel et al. 2020); (2) plant diversity cannot reduce the impact of drought on vegetation (Bonal et al. 2017; Forrester 2015; Grossiord et al. 2014a, 2014b); (3) Whether there is a diversity benefit depends on the local environment, particularly how dry the climate is (Grossiord et al. 2014a, 2014b; Jucker et al. 2016; Ratcliffe et al. 2017). The third recognition is generally accepted, while there is still some controversy about how the climate affects the diversity benefit effect. The research of Liu et al. (2022) shows that the diversity benefit is more significant in arid areas, while Jucker et al. (2014) found in a study of Iberian forests that the diversity benefit is seriously reduced under drought conditions due to the increased competition with neighboring oak trees for water. Such controversy motivated us to further explore the relationship between plant diversity and drought resistance under different climatic types.

Most of the previous studies on the relationship between plant diversity and drought resistance were conducted with in situ data at a small or point scale (Hector et al. 2010). For example, Pretzsch et al. (2013) investigated the resistance of European tree species to drought stress with tree ring measurements. Similar tree ring measurements were also utilized in studying the climate‐tree growth relationships of pure *Abies alba* in the Vosges mountains, western Europe (Lebourgeois et al. 2013). Isbell et al. (2015) collected data from 46 experiments that manipulated grassland plant diversity and measured productivity to investigate the relationship between biodiversity and the resistance of ecosystem productivity. Collecting in situ data on plant diversity and drought resistance is time‐consuming, labor‐intensive, and costly. Moreover, the spatial representation of in situ data is limited, and so is the spatial coverage of the in situ data.

In contrast, satellite remote sensing is an advanced detection technology that acquires information about our earth via remote instruments on satellite‐based platforms. Satellite remote sensing can provide a global perspective and a wealth of data about terrestrial ecosystems. Thus, exploring the relationship between plant diversity and drought resistance with satellite remote sensing could contribute to a more comprehensive and scientific understanding (Ammer 2019). With the development of remote sensing and more and more remote sensing data, the application of remote sensing has received some attention in this field. For example, Sun et al. (2013) proposed a vegetation drought index (VDI) based on remote sensing data to monitor agricultural or vegetation drought. Jalayer et al. (2023) assessed various remote sensing‐based drought indexes in investigating the spatiotemporal variations of agricultural droughts between 2000 and 2021 in Iran. Sun et al. (2024) presented a critical review of the remote sensing of vegetation drought, where the remote sensing monitoring methods of vegetation drought were divided into three categories: drought‐causing factor monitoring method, vegetation condition monitoring method, and comprehensive monitoring method. Following the idea of comprehensive monitoring, Xu et al. (2024) contributed a high spatial resolution drought response index (HiDRI) based on remote sensing data. As to the relationship between plant diversity and drought resistance, Liu et al. (2022) used the variation of remote sensing‐derived normalized difference vegetation index (NDVI) to measure vegetation drought resistance. However, the plant diversity was measured according to an in situ global tree species richness database from over 0.7 million plots. To sum up, satellite remote sensing has proven to be an effective tool for drought monitoring and evaluation. However, research on the plant diversity and drought resistance relationship was still rarely conducted with remote sensing at a larger spatial scale and longer time series, which is not conducive to understanding the role of plant diversity at multiple spatial scales (van Rooijen et al. 2015).

Therefore, an exploration of the relationship between plant diversity and drought resistance with satellite remote sensing data was conducted in this study. For this purpose, we employed a coefficient of variation (CV) method to evaluate the plant diversity of the forest ecosystem (hereafter abbreviated as forest diversity). Moreover, a remote sensing‐based standardized vegetation index (SVI) with a long time series was employed to measure the forest vegetation status, and the standardized precipitation evapotranspiration index (SPEI) was employed to express drought stress. The temporal correlation between the SVI and the SPEI time series was utilized to indicate the drought resistance of the forest ecosystem (Sun et al. 2023a). A moving window analysis method was developed to explore the relationship between forest diversity and drought resistance under different climate types. Detailed results and methods are presented in the following content. This study is significant for scientific and accurate drought management, as well as mitigating the negative effects of drought in the context of climate change.

## 2 Study Area and Data Source

### 2.1 Study Area

**FIGURE 1:**
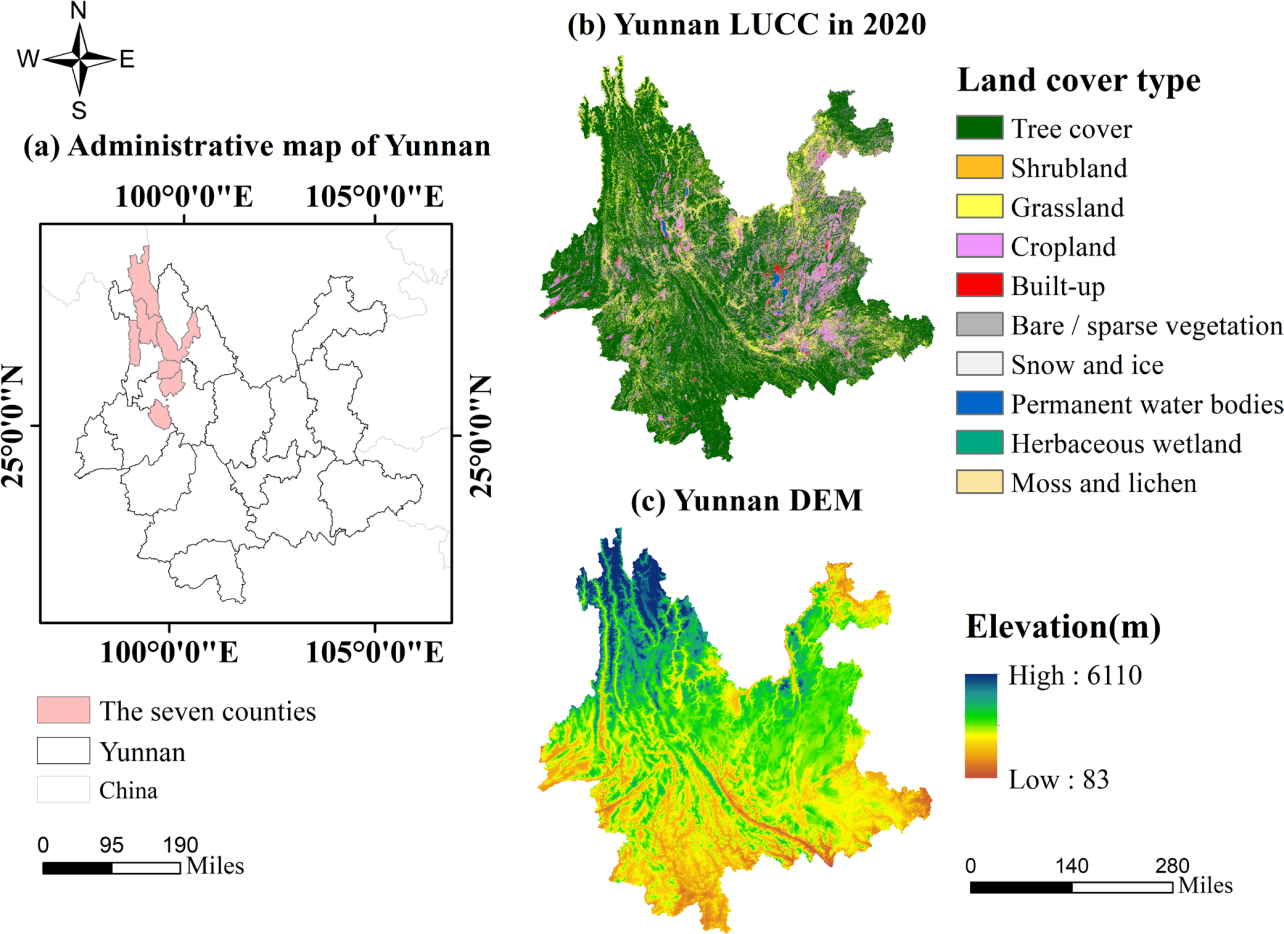
Overview of the study area where (a) is the spatial location, (b) is the land cover type in 2020, and (c) is the digital elevation model (DEM) map.

Yunnan Province of China was selected as the study area given that (1) it is a typical hotspot of drought, (2) it has a high variation of forest diversity, and (3) it has different climate types. Figure 1 shows an overview of the study area. According to the data of Yunnan Forestry and Grassland Bureau, the forest coverage reached 62.4%, the forest area reached 21.062 million hectares, the forest stock reached 2.02 billion cubic meters, and the grassland comprehensive vegetation coverage reached 87.9% in 2019. The study area's northwest has a cold climate with long winters and no summers. The study area's eastern and central parts have a temperate climate with four seasons, like spring. The study area's south belongs to the low‐heat valley area, some of which are in the south of the Tropic of Cancer and enter the tropical area, with long summers and no winter. The topography of the whole province is high in the northwest and low in the southeast, and it descends step by step from north to south. Note that this paper only explores the plant diversity benefit effect on the forest ecosystem. Therefore, the areas identified as tree land cover types by remote sensing were maintained, and those with non‐tree land cover types were excluded from the analysis.

### 2.2 Data Source

#### 2.2.1 MODIS Data

MODIS Terra MOD09A1 products were utilized to obtain remote sensing vegetation indexes (VIs) which include the normalized difference vegetation index (NDVI) and the enhanced vegetation index (EVI) every month from 2000 to 2018. The MOD09A1 data have a temporal resolution of 8 days and a spatial resolution of 500 m. First, all eligible surface spectral reflectance images of each month were selected according to the quality control band, where the pixels with a cloud state of ‘clear’ were retained and the other pixels were excluded. The 8‐day reflectance data were then aggregated into a monthly scale with the maximum value aggregation. Finally, monthly remote sensing VIs were calculated with the monthly reflectance data.

#### 2.2.2 Sentinel Data

The Sentinel‐2 satellite is equipped with a multispectral imager (MSI), which is loaded on two subsatellites. One subsatellite's revisit period is 10 days, whereas that of the two subsatellites is 5 days. The MSI has various spectral bands ranging from visible to short‐wave infrared bands and has three important bands in the red edge. The spatial resolution of MSI data varies with spectral bands at 10 m, 20 m, and 60 m. Such spatiotemporal resolution of MSI makes it very useful for distinguishing different vegetation types. Therefore, the Sentinel‐2 MSI data were used to calculate the CV index in this study.

Additionally, a Sentinel data‐based land cover type dataset named ESA WorldCover 10 m v100 in 2020 was also used to identify forest areas and non‐forest areas. This land cover type dataset has a spatial resolution of 10 m, which was produced by combining the Sentinel‐1 and Sentinel‐2 data. There are 11 land cover types in this dataset, which are tree cover, shrubland, grassland, cropland, built‐up, permanent water bodies, and so on. In this study, the tree cover type with a labeled value of 10 was taken as the forest area.

#### 2.2.3 SPEI Data

Here, the SPEI was used to measure the drought stress. It was constructed by introducing potential evapotranspiration based on the standardized precipitation index (SPI) by Vicente‐Serrano et al. (2010a); Vicente‐Serrano et al. (2010b). Numerous studies have indicated that SPEI is more appropriate than SPI to monitor drought under climate change, given the increase in evapotranspiration brought on by climate warming (Li et al. 2020). Generally, the drought event occurs when SPEI is less than −0.5. When SPEI is less than −2.0, the drought event can be recognized as an exceptional drought. The numerical variation of SPEI is continuous, and the lower the SPEI value, the more severe the drought.

The dataset of SPEI utilized in this investigation was obtained from the worldwide 0.5° SPEI grid data set (SPEIbase v2.6, https://digital.csic.es/handle/10261/202305). This dataset has a time resolution of months, provides an SPEI time scale of 1 to 48 months and covers the period from January 1901 to December 2018. It can represent long‐term and robust drought information worldwide. In this study, we utilized the SPEI data of 1‐month, 3‐month, and 6‐month time scales in every month from 2000 to 2018. To match the spatial resolution of VIs, the SPEI data were resampled into 1 km by the nearest neighbor interpolation method.

#### 2.2.4 Meteorological Data

Meteorological data were collected to calculate the aridity index (AI), which is used to measure the climate dryness of the study area. Here, the meteorological data of CRA40 (CRA's Global Atmospheric Reanalysis, CRA40, http://data.cma.cn/analysis/CRA40) were selected. It is the first generation of atmospheric reanalysis products independently developed by China, including global atmospheric reanalysis products and global land surface reanalysis products (CRA40/Land). In this study, the monthly dataset of CRA40/Land is used. The time range of the selected CRA40/Land dataset covers 31 years from 1990 to 2020, and the spatial resolution is 0.5° × 0.5°. The AI is a straightforward and practical dryness index based on long‐term climate water shortage, which is determined by the ratio of precipitation to potential evapotranspiration data. AI is a frequently used index to assess how dry the environment is in a certain area. The AI can be defined as

(1) $$\mathrm{AI}=\frac{\sum_{i=1}^{n}\left(\frac{P_{i}}{{\mathrm{PET}}_{i}}\right)}{n},$$

where i denotes the *i*th year and *n* represents the total number of years of selected precipitation and potential evapotranspiration data. In this study, *n* = 31. Like SPEI, the numerical variation of AI value is continuous. The lower the AI value, the drier the climate. In contrast, the greater the AI value, the wetter the climate. Generally, the climate type can be divided by the aridity index according to Table 1.

**TABLE 1:** Climate types that are divided by the aridity index.

Number	Climate types	Aridity index
1	Hyper‐arid	AI < 0.05
2	Arid	0.05 ≤ AI < 0.2
3	Semi‐arid	0.2 ≤ AI < 0.5
4	Dry subhumid	0.5 ≤ AI < 0.65
5	Humid	AI ≥ 0.65

## 3 Methods

### 3.1 Evaluating the Forest Diversity

Here, the CV method was employed to evaluate the plant diversity of the forest ecosystem because it is a comprehensive indicator of spectral diversity, which can be represented as a measure of plant diversity (Sun et al. 2021). First, the forest areas were identified with the land cover type dataset introduced in the above section. Second, it was assumed that there was a sliding window in the multi‐band reflectance image. The CV was first calculated using the forest vegetation pixels in this sliding window of each spectral reflectance band. Then, the average value of the CV of all bands was calculated and given to the central pixel of the sliding window. This value was considered to indicate the plant diversity of the central pixel. Subsequently, the window's continual sliding enables the calculation of the plant diversity over the whole study area. This CV method can be expressed as

(2) $$\mathrm{CV}=\sum_{\lambda =1}^{\Delta }\left(\frac{\mathrm{std}\left(\rho_{\lambda }\right)}{\text{mean}\left(\rho_{\lambda }\right)}\right)/\Delta ,$$

where $\rho_{\lambda }$ is the spectrum reflectance at the band of λ, Δ is the total number of spectral bands, std is the function to calculate the standard deviation, and mean is the function to calculate the average value.

We calculated the CV with the help of the Google Earth Engine (GEE) platform. First, we screened out all the eligible Sentinel‐2 data in 2020. The selected bands were Band2‐Blue, Band3‐Green, Band4‐Red, Band5‐Vegetation Red Edge, Band6‐Vegetation Red Edge, Band7‐Vegetation Red Edge, Band8‐NIR, Band8A‐Vegetation Red Edge, Band11‐SWIR, and Band12‐SWIR. Therefore, Δ in Equation (2) is set to 10, and the resolutions of all bands are directly resampled to 10 m in GEE. The size of the sliding window in the CV calculation was set 7 × 7 according to previous research (Sun et al. 2021). After calculating the CV results of all images in 2020, take the average image to get the final CV results. Finally, the CV result's resolution was set to 1 km when exporting the result data in GEE.

### 3.2 Measuring the Drought Resistance

The drought resistance of a vegetation ecosystem can be defined as the capacity to maintain a vegetation growth state under the disturbance of droughts. According to this definition, we employed SPEI to express the disturbance of droughts and utilized SVI to indicate the vegetation state. Subsequently, we used the Pearson correlation coefficient of monthly SVI and SPEI time series data from 2000 to 2018 to measure the drought sensitivity or drought resistance of the forest ecosystem (Sun et al. 2017, 2023a). The SVI can be expressed as the following equation:

(3) $${\mathrm{SVI}}_{i,j}=\frac{{\mathrm{VI}}_{i,j}-u_{i}}{\sigma_{i}},$$

where SVI is the standardized vegetation index, ${\mathrm{VI}}_{i,j}$ is the monthly remote sensing vegetation index (NDVI or EVI) in month i and year j, $\mu_{i}$ is the average value of ${\mathrm{VI}}_{i,j}$ in multi years, and $\sigma_{i}$ is the standard deviation of ${\mathrm{VI}}_{i,j}$ in multi years (Sun et al. 2017). Two remote sensing vegetation indexes (VIs) were used in this study to calculate SVI, which were calculated according to the following equations:

(4) $$\text{NDVI}=\frac{\text{Band}2-\text{Band}1}{\text{Band}2+\text{Band}1},$$

(5) $$\mathrm{EVI}=2.5\times \left(\frac{\text{Band}2-\text{Band}1}{\text{Band}2+6\times \text{Band}1-7.5\times \text{Band}3+1}\right),$$

where Band1, Band2, Band3, Band6, and Band7 are MOD09A1 products reflectance of corresponding bands (Sun et al. 2017). The SVIs based on NDVI and EVI were labeled as NSVI and ESVI, respectively.

Given two long time series variables SVI and SPEI at a pixel, the sensitivity or inversely the resistance of that pixel to drought disturbance (abbreviated as drought sensitivity or inversely drought resistance) can be expressed as follows:

(6) $$\gamma =\frac{\sum_{i=1}^{n}\left({\mathrm{SVI}}_{i}-\bar{\mathrm{SVI}}\right)\left({\text{SPEI}}_{i}-\bar{\text{SPEI}}\right)}{\sqrt{\sum_{i=1}^{n}{\left({\mathrm{SVI}}_{i}-\bar{\mathrm{SVI}}\right)}^{2}}\sqrt{\sum_{i=1}^{n}{\left({\text{SPEI}}_{i}-\bar{\text{SPEI}}\right)}^{2}}},$$

where γ represents the measure of drought sensitivity or drought resistance at a pixel, n is the sample size, the subscript i is the *i*th observation values, $\bar{\mathrm{SVI}}$ is the average of long‐time series SVI, and $\bar{\text{SPEI}}$ is the average of long‐time series SPEI. The γ varied between −1 and 1. Implying that the drought resistance is weaker γ, the more sensitive the forest ecosystem is to the disturbance of drought, implying that the drought resistance is weaker (Zhang et al. 2017). In contrast, the smaller the γ, the less sensitive the forest ecosystem is to the disturbance of drought, implying the stronger the drought resistance. Hereafter, the γ is employed as the metric to measure the drought resistance of forest ecosystems. It is noticeable that the forest of this study area was found more sensitive to drought in the dry seasons and less in the wet seasons according to the following analysis. Therefore, the γ between SVI and SPEI was calculated using data during the dry seasons from 2000 to 2018. In addition, the γ result's resolution was set to 1 km when exporting the result data in GEE.

### 3.3 Exploring the Relationship Between Forest Diversity and Drought Resistance

To measure the relationship between forest diversity and drought resistance, some high‐quality samples are necessary. The ‘high‐quality’ here has at least two meanings: (1) the number of samples should be enough to support the correlation analysis; (2) the samples should have the same geographical and climatic environment as much as possible. The First Law of Geography told us that “Everything is related to everything else, but near things are more related to each other”. Therefore, the geographically close samples can meet the above criteria of ‘high‐quality’. Thus, we developed a moving window analysis method to explore the relationship between forest diversity and drought resistance.

Firstly, the forest diversity image, the drought resistance image, and the climate dryness image are synthesized into a multi‐band image file with a resolution of 1 km. Then, suppose there is a moving window. In the moving window, the Pearson correlation coefficient of forest diversity and drought resistance is calculated to represent the relationship between them quantitatively. The correlation coefficient between forest diversity and drought resistance varies between −1 and 1. If the correlation coefficient is less than 0, it means that the greater the forest diversity, the stronger the drought resistance. In other words, the negative correlation coefficient indicates that there is forest diversity benefit on drought resistance. Moreover, the greater absolute value of the negative correlation coefficient indicates a stronger benefit effect. In contrast, when the correlation coefficient between CV and drought sensitivity metric is around 0 or even greater than 0, it means that there is no forest diversity benefit on drought resistance. In addition, the average value of climate dryness within a moving window was calculated to represent the average dryness of climate for that window.

Resultantly, there are two values calculated from each moving window: the correlation coefficient between plant diversity and drought resistance, and the mean value of climate dryness. With the continuous sliding of windows and there being no overlap between moving windows, more and more samples are produced over the study area. Finally, we can evaluate the relationship between plant diversity and drought resistance under different climate dryness using a scatter plot. As to the size of the moving window, it should neither be too small nor too large. If the size is too small, there are not enough samples for correlation analysis. If the size is too large, the relationship between plant diversity and drought resistance should be influenced by more geographical and climatic factors. Since the original spatial resolution of the climate dryness image, that is, the AI data, is 0.5° × 0.5°, the main window size was set to 50 pixels × 50 pixels in the result section. Besides, the sizes of 30 pixels × 30 pixels, 40 pixels × 40 pixels, and 60 pixels × 60 pixels were also analyzed in the discussion section.

## 4 Results

### 4.1 Evaluation of Forest Diversity

**FIGURE 2:**
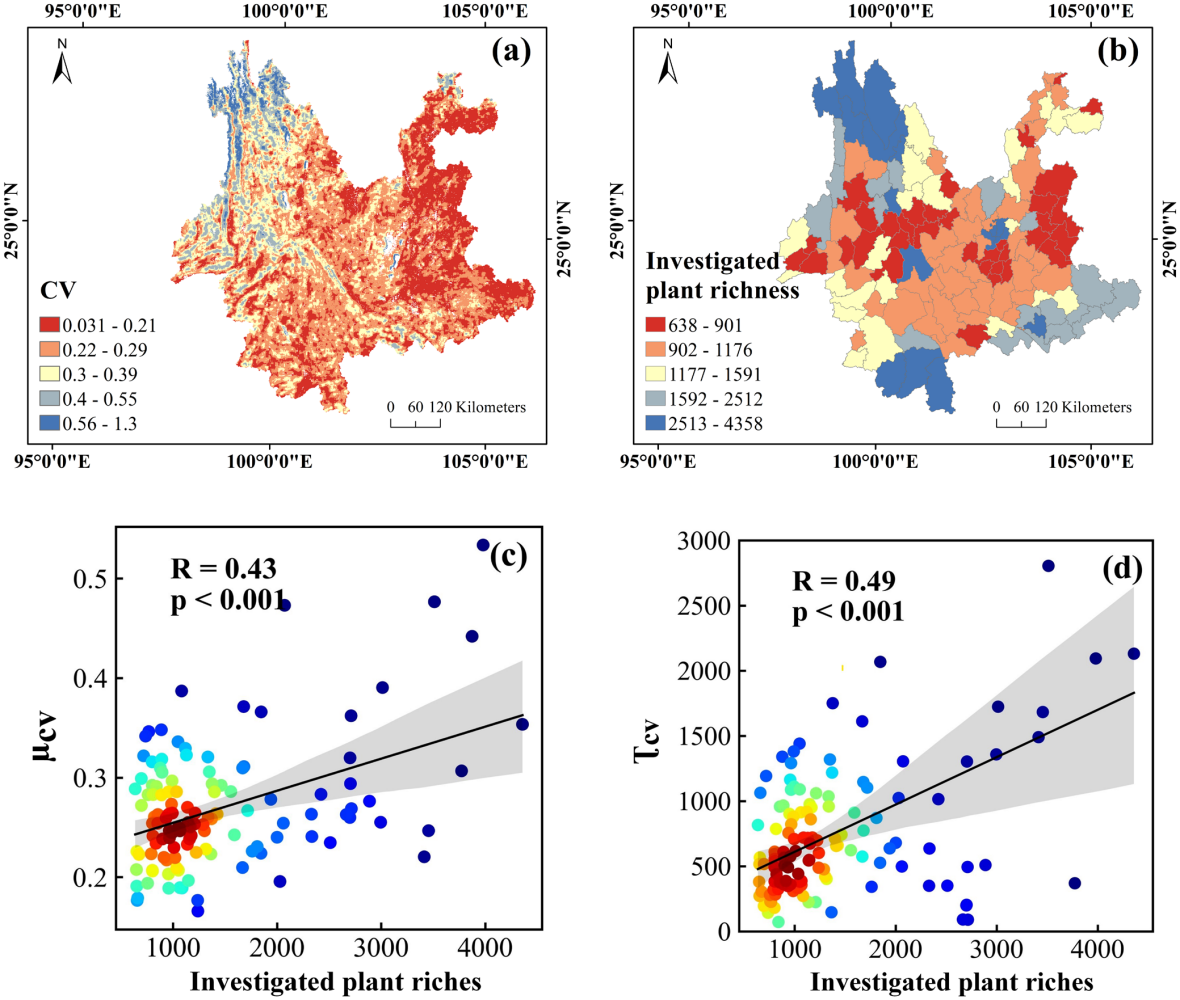
Comparison between remotely sensed forest diversity and investigated data where (a) is the CV index of forest diversity with remote sensing, (b) is the investigated data based on “Flora of Yunnan” by Li et al. (2013), (c) is the scatter plot between $\mu_{\mathrm{CV}}$ and investigated data, and (d) is the scatter plot between $\tau_{\mathrm{CV}}$ and investigated data.

Although the CV index has been validated in previous studies, it is better to compare the remote sensing result with field investigations in this study area. Here, we collected an investigated plant species dataset provided in the paper of (Li et al. 2013) according to the “Flora of Yunnan”. Subsequently, we calculated plant richness at the county level. Figure 2a,b present the forest diversity evaluated by the CV index with remote sensing data and that by the in situ investigated data, respectively. Comparisons between Figure 2a,b demonstrated that there is good consistency between the overall distribution trend of the remote sensing result and the investigated plant richness data. The northwest of Yunnan Province has the highest forest diversity, while the northeast of Yunnan Province has relatively low forest diversity. To further compare the remote sensing result with the investigated data, we made a statistic of the CV by county and analyzed the correlation between the statistics of CV within the county and the investigated data. Figure 2c,d show the comparison results with scatter plots and linear fitting. The average and sum of CV within the county (i.e., $\mu_{\mathrm{CV}}$ and $\tau_{\mathrm{CV}}$) are significantly correlated with the investigated data. The correlation between investigated data and $\tau_{\mathrm{CV}}$ is stronger than that between investigated data and $\mu_{\mathrm{CV}}$. Their correlation coefficients (*R*) are 0.49 and 0.43, respectively, with *p*‐values < 0.001. Certainly, there exists some difference between the CV result and the investigated plant richness data. The difference may be because of the uncertainties in both the remote sensing data and the investigated data. Because of the general consistency as shown in Figure 2, the classical CV method based on remote sensing is reliable for this area to express the plant diversity.

### 4.2 Evaluation of Drought Resistance

**FIGURE 3:**
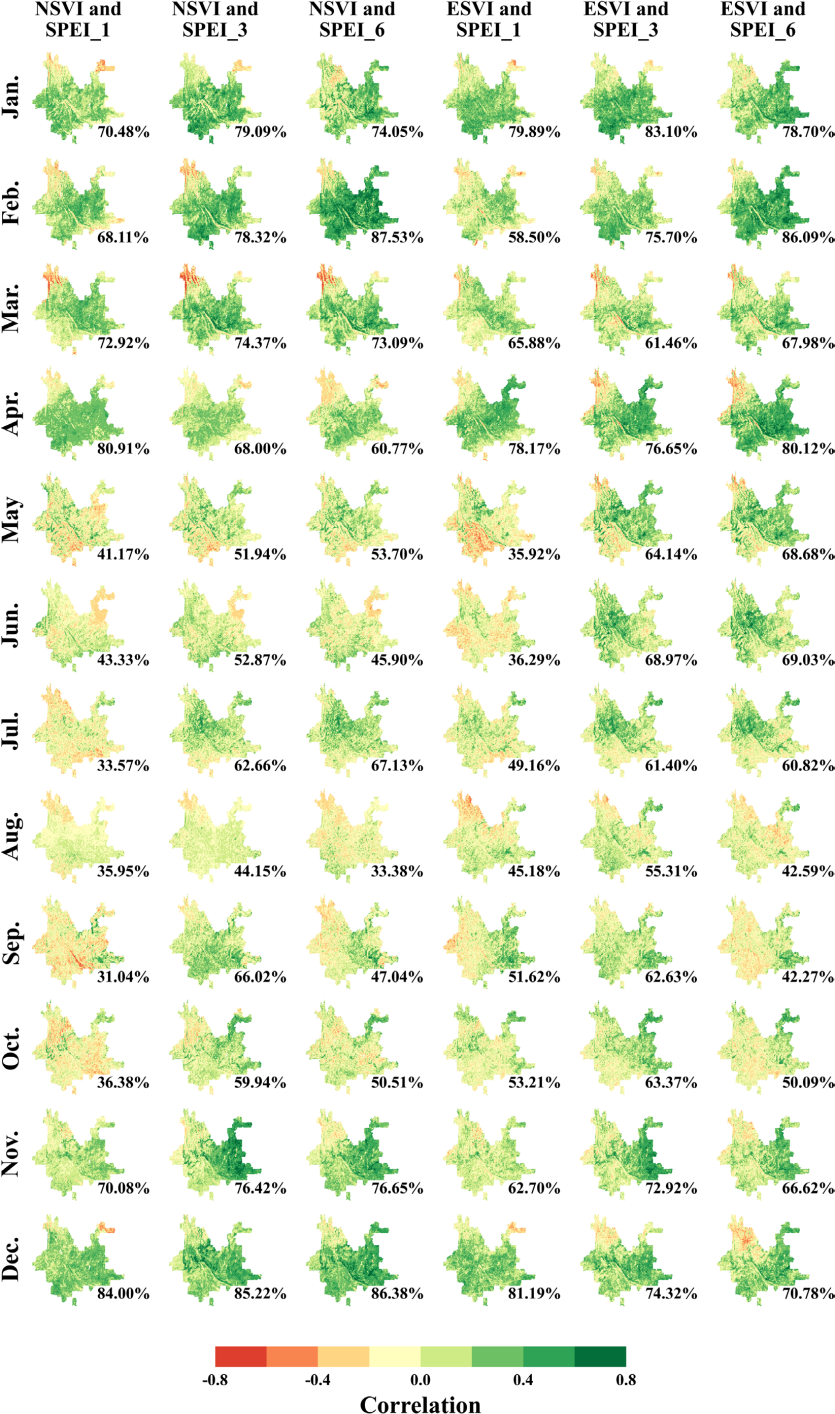
The spatial distribution of temporal correlation coefficients between NSVI/ESVI and SPEI_1/SPEI_3/SPEI_6 for each month in the study area, where the area proportion with positive correlation coefficients is marked in the lower‐right corner of each subplot.

Figure 3 shows the spatial distribution map of the correlation coefficient between SVIs (NSVI and ESVI) and SPEI_1, SPEI_3, and SPEI_6 in different months, which can indicate the drought resistance of vegetation. The positive correlation means that the forest ecosystem is sensitive to drought stress. The stronger the positive correlation, the more sensitive or the less resistant the forest ecosystem is to drought stress. In contrast, the zero or negative correlation means that the forest ecosystem is not sensitive to drought stress. The stronger the negative correlation, the more resistant the forest ecosystem is to drought stress.

Figure 3 indicates that during the dry season of the study area, that is, from January to April, and in November and December, many areas showed positive correlations. Specifically, the area proportion of having positive correlation coefficients varied between 60% and 88%, approximately, which means that most of the vegetation is sensitive to drought during these dry seasons. During the wet seasons of the study area, that is, from May to October, most areas have correlation coefficients between −0.2 and 0.2. Especially for SPEI_1, the area proportion with positive correlation coefficients varied between 30% and 50%, approximately. The results imply that most of the vegetation is insensitive to drought during this wet period. Overall, Figure 3 shows that vegetation in the study area is more sensitive to drought in the dry seasons and less sensitive in the wet seasons. Therefore, the data during the dry seasons (i.e., January to April, November, and December) from 2000 to 2018 were used to explore the relationship between plant diversity and drought resistance.

### 4.3 Relationship Between Forest Diversity and Drought Resistance

**FIGURE 4:**
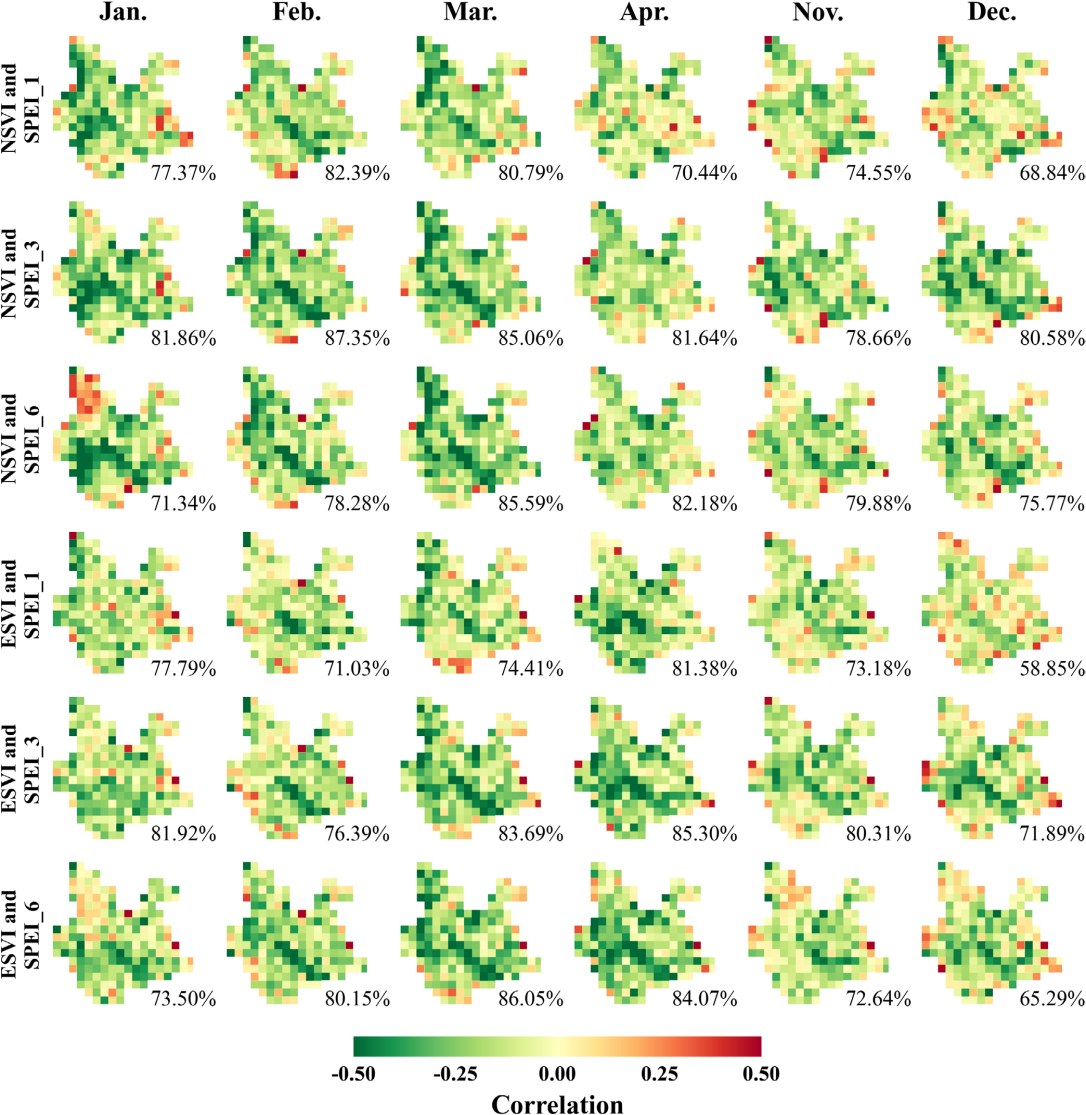
Maps of the correlation coefficient between plant diversity and drought resistance, where the lower right corner gives the percentage of area with a negative correlation coefficient.

Figure 4 shows the spatial distributions of correlation coefficients between plant diversity and drought resistance obtained by the moving window analysis as described in section 3.3. The drought resistance data used in the first three lines are based on the correlations between NSVI and SPEI_1, SPEI_3, and SPEI_6 in different months. The drought resistance data used in the last three lines are based on the correlations between ESVI and SPEI_1, SPEI_3, and SPEI_6 in different months. The value of each pixel represents the relationship between plant diversity and drought resistance within each moving window. The lower right corner gives the area proportion of having a negative correlation coefficient. The negative correlation coefficient means that the higher the forest diversity, the less the drought sensitivity, or the stronger the drought resistance. As can be seen from Figure 4, the area proportion of having a negative correlation coefficient varied around 80%, which means that the forest diversity was positively correlated with drought resistance in most areas. In other words, the forest diversity presented a beneficial effect on forest drought resistance in most of the study area.

**FIGURE 5:**
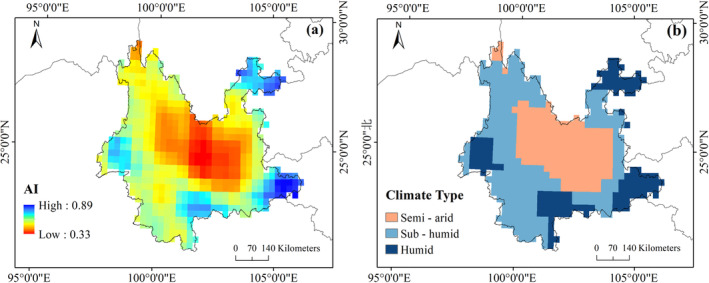
(a) Spatial distribution of the AI map in Yunnan Province, and (b) climatic types of Yunnan Province divided by AI.

In addition, the deepest green area in Figure 4 is generally located in the central part of the study area. Figure 5 shows the spatial distribution of AI and the climate types according to AI classification. There are three main climate types in Yunnan Province, that is, semi‐arid, dry subhumid, and humid. The central part of the study area belongs to a semi‐arid climate. The semi‐arid climate areas are surrounded by the dry subhumid climate areas. The humid climate areas are distributed within the edge of the study area. Figures 4 and 5 imply that the beneficial effect of forest diversity is stronger in relatively arid areas.

**FIGURE 6:**
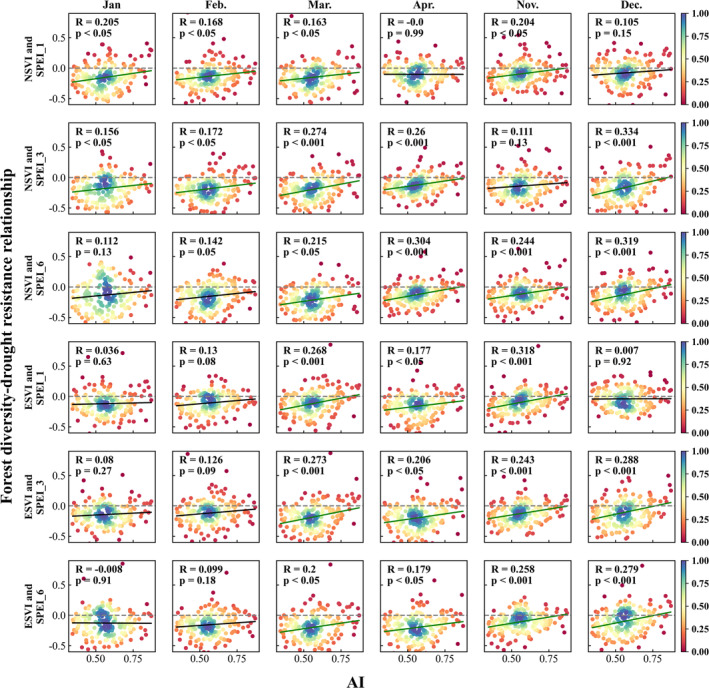
The scatter density plot of the relationship between forest diversity and drought sensitivity varied with the AI values.

Figure 6 shows the scatter density plot obtained by the moving window analysis method. It shows how the relationship between forest diversity and drought sensitivity changes with the variation of the climate dryness. The vertical axis represents the correlation coefficients between forest diversity and drought sensitivity. The horizontal axis represents the values of AI. The first three rows are based on the correlation coefficients between NSVI and SPEI_1, SPEI_3, and SPEI_6 in different months, respectively. The last three rows are based on the correlation coefficients between ESVI and SPEI_1, SPEI_3, and SPEI_6 in different months, respectively. Most of the correlation coefficients are less than zero, which indicates that plant diversity does have a beneficial effect on promoting drought resistance in this study area. Figure 6 also presents a significant increasing trend of the correlation coefficient with the increase of AI. The climate gets drier as AI gets smaller. The climate becomes increasingly humid as AI increases. Therefore, we can conclude that the beneficial effect of forest diversity is stronger in more arid areas than in humid areas.

## 5 Discussion

### 5.1 The Influence of Another Forest Diversity Index on the Results

**FIGURE 7:**
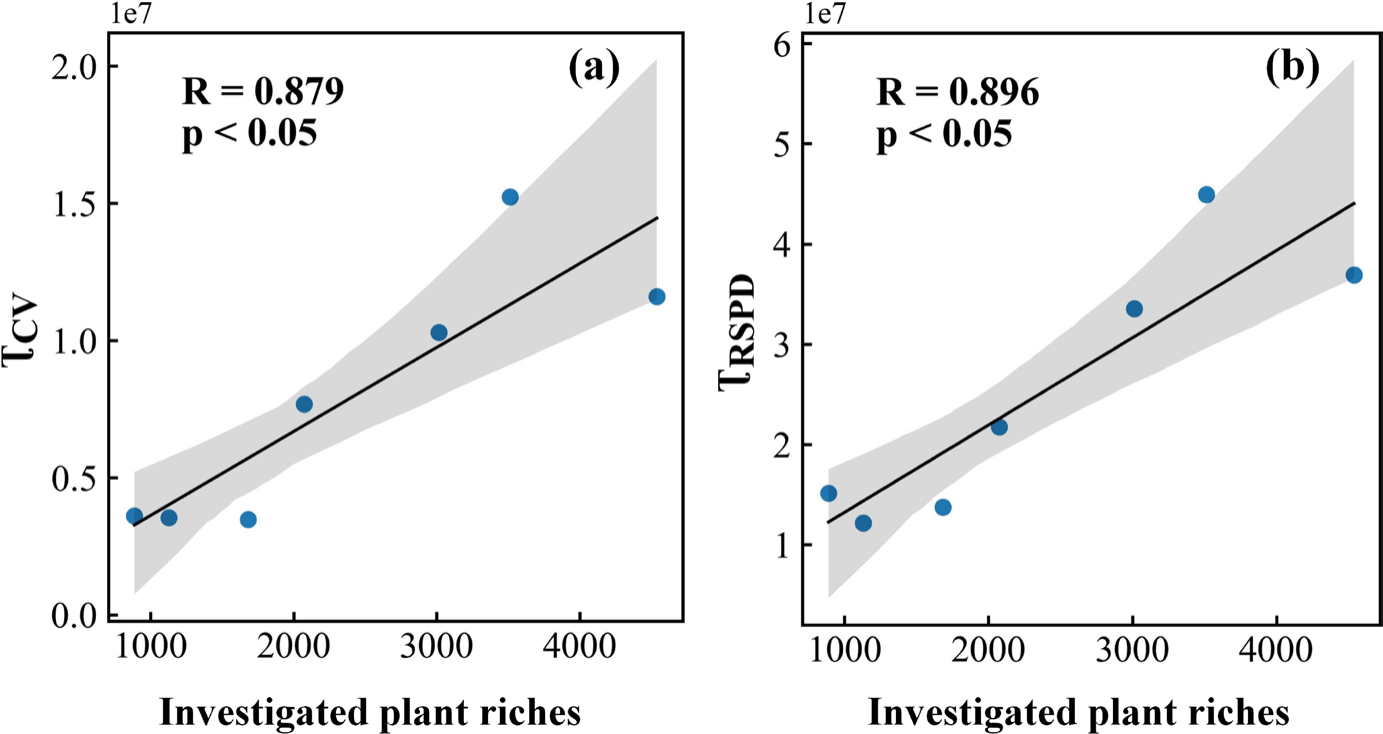
The correlation between the investigated plant richness and (a) the sum of CV ($\tau_{\mathrm{CV}}$) and (b) the sum of RSPD ($\tau_{\text{RSPD}}$) in seven typical counties.

**FIGURE 8:**
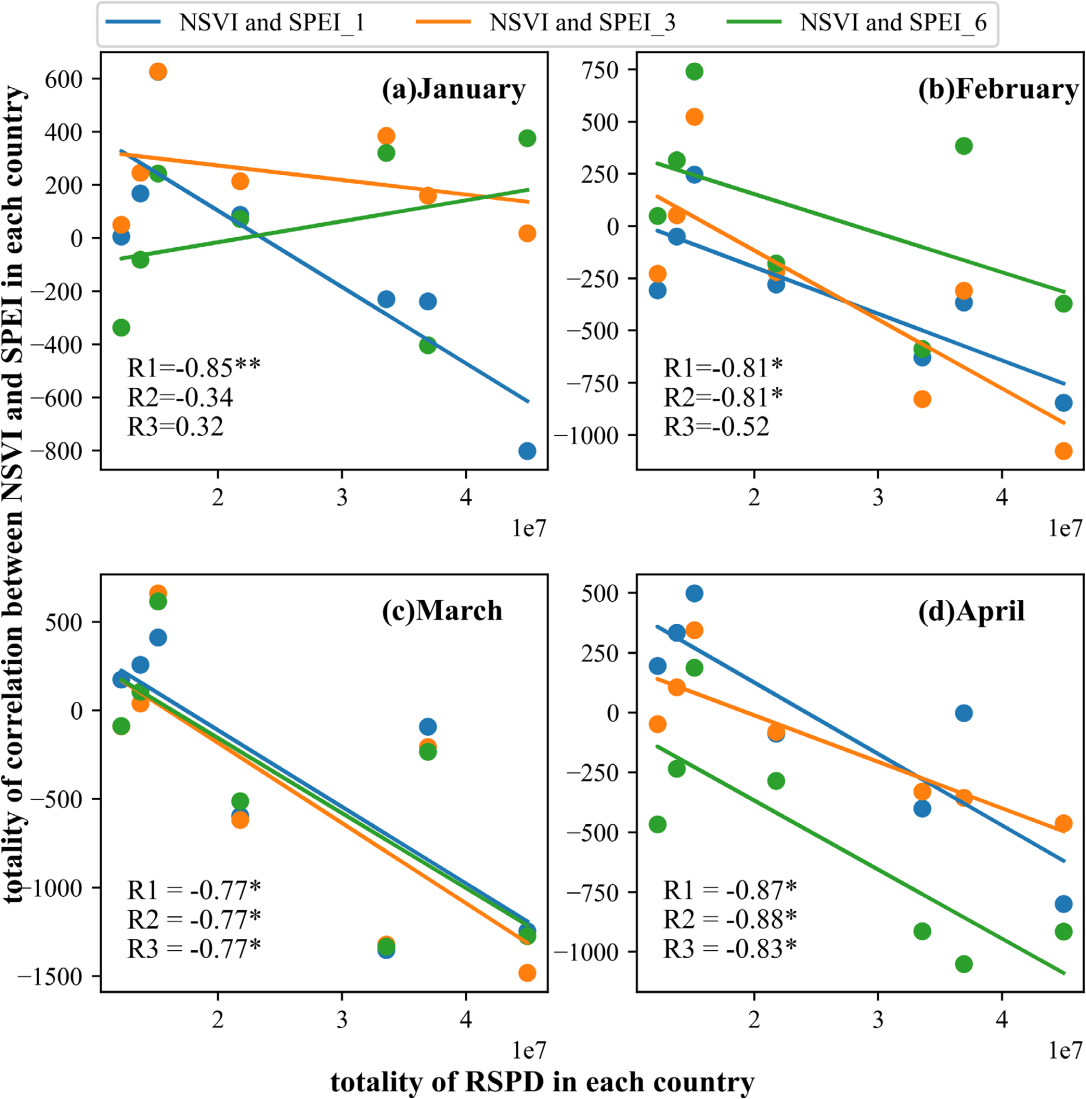
Scatter plot of the relationship between the totality of drought sensitivity and the totality of RSPD in seven typical counties, where * represents *p* value < 0.05, and ** represents *p* value < 0.001.

The RSPD index (a remote sensing index of plant diversity) is a relatively new plant diversity index constructed by Sun et al. (2021) based on the combination of the spectral variation hypothesis and the productivity hypothesis. However, because the calculation efficiency of RSPD is not as high as that of CV, the CV was selected in the above analysis. To discuss the influence of another forest diversity index on the previous results, the RSPD index was used here to explore the relationship between plant diversity and drought resistance. Because of the relatively low calculation efficiency of RSPD, the RSPD was evaluated in seven counties from January to April. The seven counties are Deqin County, Yulong County, Jianchuan County, Weixi County, Eryuan County, Fugong County, and Yongping County; the distribution of the seven counties is shown in Figure 1. Figure 7 shows the scatter plot of CV, RSPD, and investigated plant richness in the seven counties. The results show that both CV and RSPD are significantly positively correlated with the investigated data. RSPD has a stronger positive correlation, indicating that RSPD has a better effect than CV on expressing plant diversity. However, using a CV to represent regional plant diversity has a similar performance to that of RSPD. Figure 8 shows the comparisons between the totality of RSPD in each county and the totality of drought resistance in each county. Figure 8 illustrates that the greater the RSPD, the stronger the drought resistance. The evaluations with RSPD also demonstrate that plant diversity promotes drought resistance, which implies that the results identified by CV would not be overturned by another forest diversity index, RSPD.

### 5.2 The Influence of Drought Frequency on the Results

**FIGURE 9:**
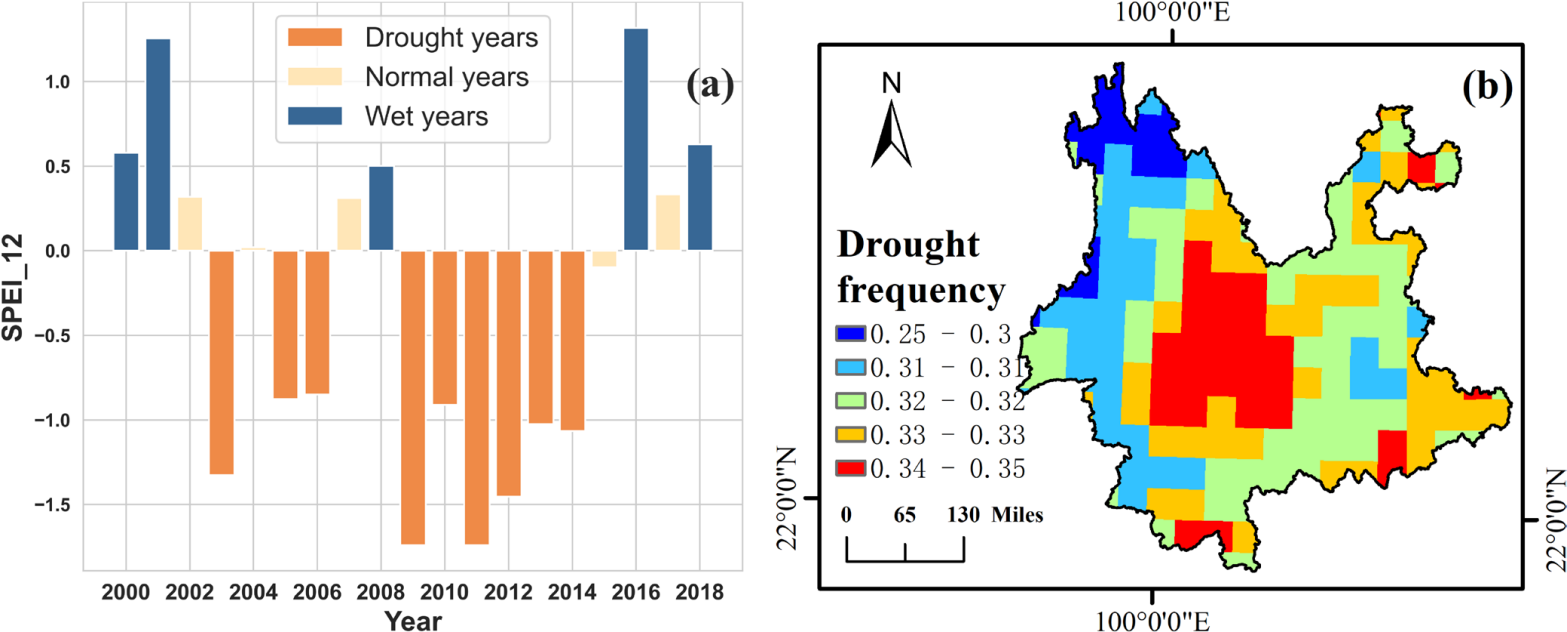
Drought features of the study area recognized by the SPEI_12 where (a) is the drought years and (b) is the drought frequency.

The study period spans from 2000 to 2018, as we have explained. Some of these years were moist, while others suffered severe drought events. To simply illustrate the drought events, SPEI at a 12‐month scale (SPEI_12) was utilized. Figure 9a shows the SPEI_12 in December of each year, which shows that 2003, 2005, 2006, and 2009–2014 are drought years where extreme drought occurred in 2009 and 2011. The years 2000, 2001, 2016, and 2018 are wet years. The other 6 years are normal. Therefore, there are nine drought years, six normal years, and four wet years in the study period. It is reasonable to explore the forest diversity–drought resistance relationship using the SPEI data during the period from 2000 to 2018.

**FIGURE 10:**
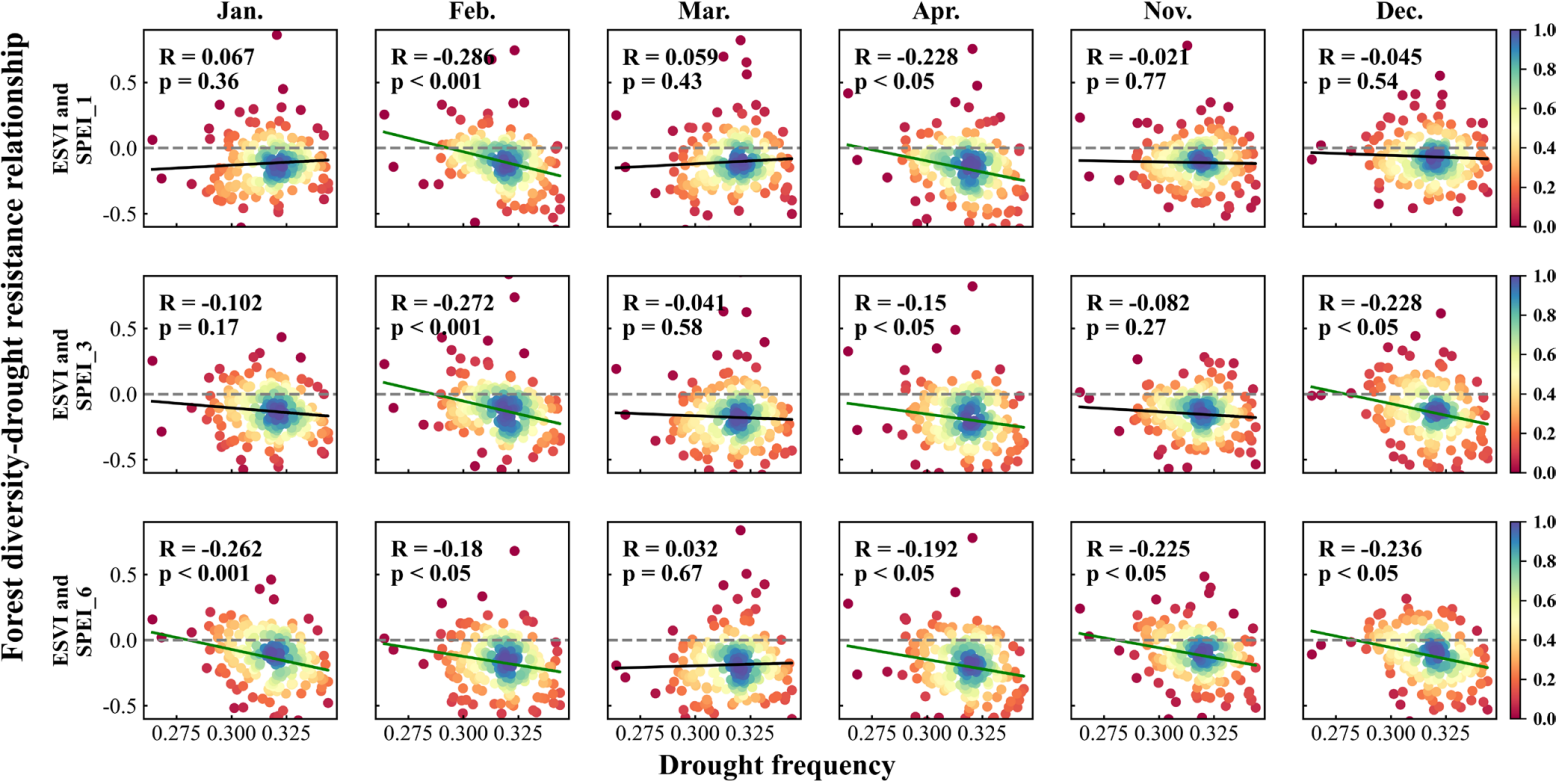
The scatter density plot of the relationship between forest diversity and drought sensitivity varied with the drought frequency.

The drought frequency in the study area was also calculated, as shown in Figure 9b, where drought frequency was calculated as the ratio of drought months to the totality of all months during the study period from 2000 to 2018. Likewise, the SPEI_12 was used to calculate the drought frequency. If the value of SPEI_12 in a month is less than −0.5, that month is then recognized as a drought month. Figure 9b demonstrates that the central part of the study area is the drought‐prone region, which is also the Semi‐arid climate zone identified by the AI index. To explore whether the beneficial effect of forest diversity on drought resistance is stronger in drought‐prone areas, the moving window analysis method, as shown in section 3.3, was used again to analyze the relationship between forest diversity and drought resistance in different drought‐prone areas. Figure 10 shows the scattering density map obtained by the moving window analysis method. The horizontal axis represents the average drought frequency in a sliding window, and the vertical axis represents the correlation coefficient between forest diversity and drought sensitivity in the same sliding window. First, most of the points have a correlation coefficient of less than 0, which means that there exists a beneficial effect of forest diversity on drought resistance. Second, the forest diversity–drought sensitivity relationship varied negatively with the increasing drought frequency. In other words, the greater the drought frequency, the stronger the negative correlation coefficient of the forest diversity–drought sensitivity relationship. Such results demonstrated that the beneficial effect of forest diversity on drought resistance is stronger in drought‐prone areas.

### 5.3 The Influence of Moving Window Size

**FIGURE 11:**
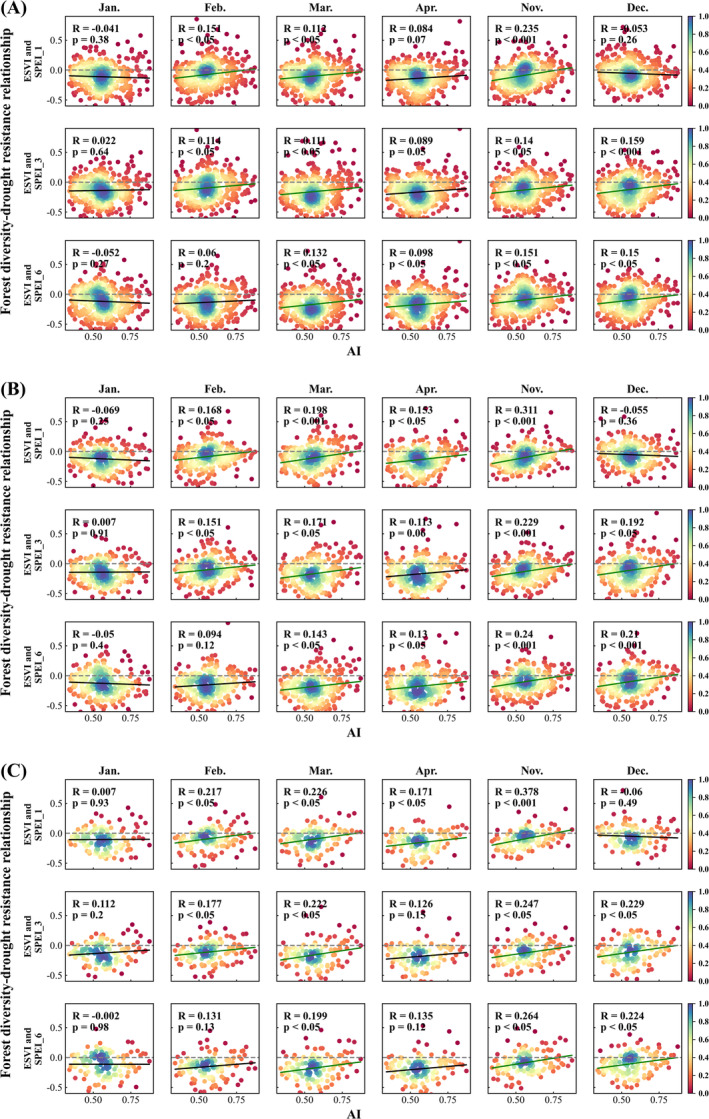
The scatter density plot of the relationship between forest diversity and drought sensitivity varying with AI under different moving window sizes, where the sizes in (A), (B), and (C) are 30 × 30, 40 × 40, and 60 × 60, respectively.

This section further explores the relationship between forest diversity and drought resistance under different sizes of the moving window, which has been mentioned in Section 3.3. Figure 11A–C shows how the relationship between forest diversity and drought resistance changes with the variation of the climate dryness using the moving window sizes of 30 × 30, 40 × 40, and 60 × 60, respectively.

Figure 11 also demonstrates that (1) most of the correlation coefficients are less than zero and (2) there is a significant increasing trend of the correlation coefficient with the increase of AI. The findings are consistent with that under the moving window size of 50 × 50 as shown in Figure 6. They all indicate that forest diversity has a beneficial effect on promoting drought resistance in this study area. Moreover, the forest diversity benefit effect is stronger in more arid areas.

### 5.4 Implications of This Study

The relationship between forest diversity and drought resistance under different climatic types was investigated in this study using satellite remote sensing. It was found that there is a wide negative correlation between forest diversity and drought sensitivity, which means that plant diversity has a significant beneficial effect on promoting the drought resistance of the forest ecosystem. In addition, we found that the beneficial effect of forest diversity is more significant in relatively arid climate zones as well as in drought‐prone areas. Our results are consistent with an investigation with in situ observations by Liu et al. (2022). They investigated more than 700,000 forest plots around the world. They found that high species diversity can significantly improve the drought resistance of about half of the global forests, but it is highly variable in space. Moreover, drought and drought‐prone forests have greater diversity benefits on drought resistance. However, it is time‐consuming, labor‐intensive, and very expensive to conduct the study with in situ observations, especially in large or continental areas. This study contributes a low‐cost and spatial method to investigate the relationship between forest diversity and drought resistance using satellite remote sensing. Such a method would have greater application potential to further explore the mechanism, uncertainty, influencing factors, and scale effect behind the forest diversity –drought resistance relationship.

**FIGURE 12:**
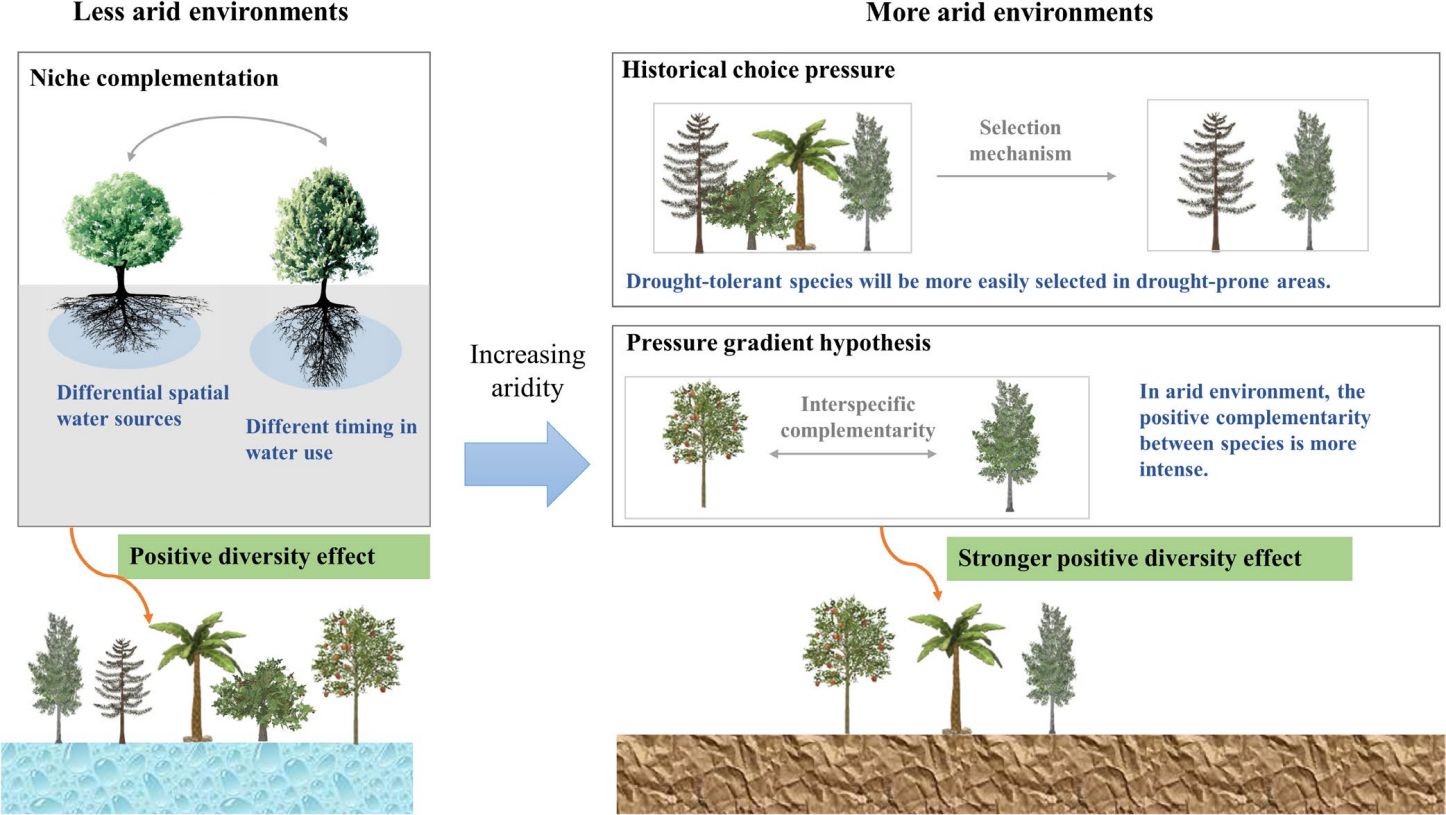
An interpretation of the relationship between plant diversity and drought resistance.

In addition to the valuable method, it is interesting to discuss the reasons behind the beneficial effect of forest diversity. Figure 12 presents the possible reasons. The following analysis focuses on two aspects: why diversity positively impacts drought resistance and why this effect is more pronounced in more arid regions.

First, the benefits of forest diversity for drought resistance may be attributed to the following mechanisms: (1) Insurance hypothesis. According to the insurance hypothesis, biodiversity ensures that the ecosystem function will not decline because many species provide stronger guarantees, and some species will maintain this function even if other species fail (Yachi and Loreau 1999). According to the insurance hypothesis, more diverse communities may be more drought‐resistant (Mariotte et al. 2013). (2) Niche complementarity. Niche complementarity can be further used to interpret the insurance hypothesis (Hao et al. 2020). This concept means that the niches of different organisms in the ecological environment are differentiated and complementary. Specifically, there are differences in time and space in resource utilization. Niche complementarity includes time niche complementarity and space niche complementarity. Time niche complementarity means that the temporal stability of ecosystem productivity increases with the increase of plant diversity due to the asynchronous response of different species to drought (Morin et al. 2014). The time asynchronism of species increases the stability of local communities, and the asynchronism between local communities improves the stability of meta‐communities to a large extent (Wilcox et al. 2017). Spatial niche complementarity refers to the interspecific differences of different species that make tree species distribute resources and complement each other in mixed forests, which makes mixed forests more productive than single cultivation (Lu et al. 2018). Some scholars have compared the relationship between the time stability and asynchrony of species growth in mixed and single species and found that the main driving factor of the stability process may be the time niche complementarity between species, rather than the difference in the internal response of species to environmental conditions; that is, the spatial niche complementarity (del Rio et al. 2017).

Second, the stronger positive effects of forest diversity on drought resistance in arid and drought‐prone areas may be explained by the following mechanisms: (1) Historical selection pressure. Through historical selection pressure, drought‐tolerant species will be more easily selected in drought‐prone areas, and the increase in forest diversity will increase the probability of finding species that are very suitable for arid conditions (Lloret et al. 2007). (2) Pressure gradient hypothesis. The pressure gradient hypothesis suggests that in harsh environments such as extreme drought, biological populations are more likely to exhibit mutually reinforcing interspecific relationships, thereby reducing water stress through positive complementarity (He and Bertness 2014). Certainly, there may be other reasons for interpreting the plant diversity benefit effect on drought resistance. This study mainly contributes a method of exploring the plant diversity–drought resistance relationship with satellite remote sensing. This method should be further applied in a wider area and more scale to enhance our understanding of the plant diversity–drought resistance relationship.

## 6 Conclusion

The relationship between plant diversity and drought resistance of forest ecosystems under different climate conditions was investigated in this study with satellite remote sensing technology. Results indicated that:

1. The forest diversity of the study area can be recognized with the Sentinel‐2 remote sensing data since the remotely sensed CV index was found significantly correlated with field investigations of forest diversity in the study area with a correlation coefficient of around 0.43–0.49 and *p* value < 0.001.
2. The forest vegetation in the study area is more sensitive to drought in the dry seasons and less sensitive in the wet seasons since the area proportion of having positive temporal correlation coefficients between SVIs and one‐month SPEI varied around 60%–88% during the dry seasons, while that varied around 30%–60% during the wet seasons. The temporal correlation between SVIs and SPEIs during the dry season was then used to measure drought sensitivity or drought resistance of forest ecosystems.
3. A moving window analysis method was developed to explore the relationship between forest diversity and drought resistance. We found that about 80% of the study area presented a beneficial effect of forest diversity on drought resistance, that is, the higher the forest diversity, the lower the forest sensitivity to drought.
4. The beneficial effect of forest diversity on drought resistance changes with the variation of climate types. It was found to be stronger in more arid and drought‐prone areas.

To sum up, this study provided evidence from satellite remote sensing that forest diversity has a beneficial effect on reducing the sensitivity of forest ecosystems to drought stress, or in other words, promoting drought resistance. Moreover, this beneficial effect was found to be more significant in relatively dry climates and drought‐prone areas. Our research results have a certain guiding significance for ecosystem management, protection, and restoration. Certainly, the remote sensing of forest diversity used in this study has limitations, such as being prone to cloud contamination and being difficult to perceive vegetation below the forest canopy. In the future, breakthroughs are required in the assessment of forest diversity by remote sensing. Moreover, more efforts are required to explore the plant diversity effect on the land surface ecosystem using the method contributed by this study at multiple scales and wider areas.

## Author Contributions

**Guotao Ma:** writing – review and editing (lead). **Hao Sun:** conceptualization (equal), formal analysis (equal), funding acquisition (equal), methodology (equal), validation (equal), writing – original draft (equal). **Keke Hu:** formal analysis (equal), methodology (equal), validation (equal), writing – original draft (equal). **Hong Zhou:** formal analysis (equal), funding acquisition (equal), visualization (equal), writing – original draft (equal).

## Conflicts of Interest

The authors declare no conflicts of interest.

## Data Availability

The data supporting the findings of this research are publicly available at https://doi.org/10.5067/MODIS/MOD09A1.061, https://zenodo.org/records/5571936, https://digital.csic.es/handle/10261/202305, http://data.cma.cn/analysis/CRA40.

## References

[ece370999-bib-0001] Ammer, C. 2019. “Diversity and Forest Productivity in a Changing Climate.” New Phytologist 221, no. 1: 50–66. 10.1111/nph.15263. 29905960

[ece370999-bib-0002] Bonal, D. , M. Pau , M. Toigo , A. Granier , and T. Perot . 2017. “Mixing Oak and Pine Trees Does Not Improve the Functional Response to Severe Drought in Central French Forests.” Annals of Forest Science 74, no. 4: 72. 10.1007/s13595-017-0671-9.

[ece370999-bib-0003] del Rio, M. , H. Pretzsch , R. Ruiz‐Peinado , et al. 2017. “Species Interactions Increase the Temporal Stability of Community Productivity in *Pinus Sylvestris*‐*Fagus Sylvatica* Mixtures Across Europe.” Journal of Ecology 105, no. 4: 1032–1043. 10.1111/1365-2745.12727.

[ece370999-bib-0004] Forrester, D. I. 2015. “Transpiration and Water‐Use Efficiency in Mixed‐Species Forests Versus Monocultures: Effects of Tree Size, Stand Density and Season.” Tree Physiology 35, no. 3: 289–304. 10.1093/treephys/tpv011. 25732385

[ece370999-bib-0005] Grossiord, C. 2020. “Having the Right Neighbors: How Tree Species Diversity Modulates Drought Impacts on Forests.” New Phytologist 228, no. 1: 42–49. 10.1111/nph.15667. 30585635

[ece370999-bib-0006] Grossiord, C. , A. Granier , A. Gessler , T. Jucker , and D. Bonal . 2014a. “Does Drought Influence the Relationship Between Biodiversity and Ecosystem Functioning in Boreal Forests?” Ecosystems 17, no. 3: 394–404. 10.1007/s10021-013-9729-1.

[ece370999-bib-0007] Grossiord, C. , A. Granier , S. Ratcliffe , et al. 2014b. “Tree Diversity Does Not Always Improve Resistance of Forest Ecosystems to Drought.” Proceedings of the National Academy of Sciences of the United States of America 111, no. 41: 14812–14815. 10.1073/pnas.1411970111. 25267642 PMC4205672

[ece370999-bib-0008] Hao, M. H. , C. Messier , Y. Geng , C. Y. Zhang , X. H. Zhao , and K. von Gadow . 2020. “Functional Traits Influence Biomass and Productivity Through Multiple Mechanisms in a Temperate Secondary Forest.” European Journal of Forest Research 139, no. 6: 959–968. 10.1007/s10342-020-01298-0.

[ece370999-bib-0009] He, Q. , and M. D. Bertness . 2014. “Extreme Stresses, Niches, and Positive Species Interactions Along Stress Gradients.” Ecology 95, no. 6: 1437–1443. 10.1890/13-2226.1. 25039207

[ece370999-bib-0010] Hector, A. , Y. Hautier , P. Saner , et al. 2010. “General Stabilizing Effects of Plant Diversity on Grassland Productivity Through Population Asynchrony and Overyielding.” Ecology 91, no. 8: 2213–2220. 10.1890/09-1162.1. 20836442

[ece370999-bib-0011] Isbell, F. , D. Craven , J. Connolly , et al. 2015. “Biodiversity Increases the Resistance of Ecosystem Productivity to Climate Extremes.” Nature 526, no. 7574: 574–577. 10.1038/nature15374. 26466564

[ece370999-bib-0012] Jalayer, S. , A. Sharifi , D. Abbasi‐Moghadam , A. Tariq , and S. Qin . 2023. “Assessment of Spatiotemporal Characteristic of Droughts Using in Situ and Remote Sensing‐Based Drought Indices.” IEEE Journal of Selected Topics in Applied Earth Observations and Remote Sensing 16: 1483–1502. 10.1109/JSTARS.2023.3237380.

[ece370999-bib-0013] Jucker, T. , D. Avăcăriței , I. Bărnoaiea , et al. 2016. “Climate Modulates the Effects of Tree Diversity on Forest Productivity.” Journal of Ecology 104, no. 2: 388–398. 10.1111/1365-2745.12522.

[ece370999-bib-0014] Jucker, T. , O. Bouriaud , D. Avacaritei , et al. 2014. “Competition for Light and Water Play Contrasting Roles in Driving Diversity‐Productivity Relationships in Iberian Forests.” Journal of Ecology 102, no. 5: 1202–1213. 10.1111/1365-2745.12276.

[ece370999-bib-0015] Lebourgeois, F. , N. Gomez , P. Pinto , and P. Merian . 2013. “Mixed Stands Reduce *Abies Alba* Tree‐Ring Sensitivity to Summer Drought in the Vosges Mountains, Western Europe.” Forest Ecology and Management 303: 61–71. 10.1016/j.foreco.2013.04.003.

[ece370999-bib-0016] Li, C. , D. Hongjin , and P. Hua . 2013. “Diversity and Distribution of Higher Plants in Yunnan, China.” Biodiversity Science 21, no. 3: 359–363. 10.3724/sp.J.1003.2013.05162.

[ece370999-bib-0017] Li, L. , D. She , H. Zheng , P. Lin , and Z.‐L. Yang . 2020. “Elucidating Diverse Drought Characteristics From Two Meteorological Drought Indices (SPI and SPEI) in China.” Journal of Hydrometeorology 21, no. 7: 1513–1530. 10.1175/jhm-d-19-0290.1.

[ece370999-bib-0018] Liu, D. , T. Wang , J. Peñuelas , and S. Piao . 2022. “Drought Resistance Enhanced by Tree Species Diversity in Global Forests.” Nature Geoscience 15: 800–804. 10.1038/s41561-022-01026-w.

[ece370999-bib-0019] Lloret, F. , A. Lobo , H. Estevan , P. Maisongrande , J. Vayreda , and J. Terradas . 2007. “Woody Plant Richness and NDVI Response to Drought Events in Catalonian (Northeastern Spain) Forests.” Ecology 88, no. 9: 2270–2279. 10.1890/06-1195.1. 17918405

[ece370999-bib-0020] Lu, H. , G. M. J. Mohren , M. del Rio , M.‐J. Schelhaas , M. Bouwman , and F. J. Sterck . 2018. “Species Mixing Effects on Forest Productivity: A Case Study at Stand‐, Species‐ and Tree‐Level in The Netherlands.” Forests 9, no. 11: 713. 10.3390/f9110713.

[ece370999-bib-0021] Mariotte, P. , C. Vandenberghe , P. Kardol , F. Hagedorn , A. Buttler , and S. Schwinning . 2013. “Subordinate Plant Species Enhance Community Resistance Against Drought in Semi‐Natural Grasslands.” Journal of Ecology 101, no. 3: 763–773. 10.1111/1365-2745.12064.

[ece370999-bib-0022] Morin, X. , L. Fahse , C. de Mazancourt , M. Scherer‐Lorenzen , and H. Bugmann . 2014. “Temporal Stability in Forest Productivity Increases With Tree Diversity due to Asynchrony in Species Dynamics.” Ecology Letters 17, no. 12: 1526–1535. 10.1111/ele.12357. 25212251

[ece370999-bib-0023] Pretzsch, H. , G. Schutze , and E. Uhl . 2013. “Resistance of European Tree Species to Drought Stress in Mixed Versus Pure Forests: Evidence of Stress Release by Inter‐Specific Facilitation.” Plant Biology 15, no. 3: 483–495. 10.1111/j.1438-8677.2012.00670.x. 23062025

[ece370999-bib-0024] Ratcliffe, S. , C. Wirth , T. Jucker , et al. 2017. “Biodiversity and Ecosystem Functioning Relations in European Forests Depend on Environmental Context.” Ecology Letters 20, no. 11: 1414–1426. 10.1111/ele.12849. 28925074

[ece370999-bib-0025] Steckel, M. , M. del Rio , M. Heym , et al. 2020. “Species Mixing Reduces Drought Susceptibility of Scots Pine (*Pinus sylvestris* L.) and Oak (*Quercus robur* L., Quercus Petraea (Matt.) Liebl.) – Site Water Supply and Fertility Modify the Mixing Effect.” Forest Ecology and Management 461: 117908. 10.1016/j.foreco.2020.117908.

[ece370999-bib-0026] Sun, H. , J. Gao , T. Yan , et al. 2024. “Remote Sensing of Vegetation Drought: Research Progress (In Chineses).” National Remote Sensing Bulletin 28, no. 6: 1395–1411. 10.11834/jrs.20243374.

[ece370999-bib-0027] Sun, H. , J. Hu , J. Wang , J. Zhou , L. Lv , and J. Nie . 2021. “RSPD: A Novel Remote Sensing Index of Plant Biodiversity Combining Spectral Variation Hypothesis and Productivity Hypothesis.” Remote Sensing 13, no. 15: 3007. 10.3390/rs13153007.

[ece370999-bib-0028] Sun, H. , W. Liu , Y. Wang , and S. Yuan . 2017. “Evaluation of Typical Spectral Vegetation Indices for Drought Monitoring in Cropland of the North China Plain.” IEEE Journal of Selected Topics in Applied Earth Observations and Remote Sensing 10, no. 12: 5404–5411. 10.1109/jstars.2017.2734800.

[ece370999-bib-0029] Sun, H. , Q. Xu , Y. Wang , et al. 2023b. “Agricultural Drought Dynamics in China During 1982–2020: A Depiction With Satellite Remotely Sensed Soil Moisture.” GIScience & Remote Sensing 60, no. 1: 2257469. 10.1080/15481603.2023.2257469.

[ece370999-bib-0030] Sun, H. , Z. Xu , and H. Liu . 2023a. “An Evaluation of the Response of Vegetation Greenness, Moisture, Fluorescence, and Temperature‐Based Remote Sensing Indicators to Drought Stress.” Journal of Hydrology 625: 130125. 10.1016/j.jhydrol.2023.130125.

[ece370999-bib-0031] Sun, H. , X. Zhao , Y. Chen , A. Gong , and J. Yang . 2013. “A New Agricultural Drought Monitoring Index Combining MODIS NDWI and Day‐Night Land Surface Temperatures: A Case Study in China.” International Journal of Remote Sensing 34, no. 24: 8986–9001. 10.1080/01431161.2013.860659.

[ece370999-bib-0032] Tsegai, D. , M. Medel , P. Augenstein , et al. 2022. “Drought in Numbers 2022.” Abidjan, Côte d'Ivoire.

[ece370999-bib-0033] Van den Berge, J. , K. Naudts , H. J. De Boeck , R. Ceulemans , and I. Nijs . 2014. “Do Interactions With Neighbours Modify the Above‐Ground Productivity Response to Drought? A Test With Two Grassland Species.” Environmental and Experimental Botany 105: 18–24. 10.1016/j.envexpbot.2014.04.002.

[ece370999-bib-0034] van Rooijen, N. M. , W. de Keersmaecker , W. A. Ozinga , et al. 2015. “Plant Species Diversity Mediates Ecosystem Stability of Natural Dune Grasslands in Response to Drought.” Ecosystems 18, no. 8: 1383–1394. 10.1007/s10021-015-9905-6.

[ece370999-bib-0035] Vicente‐Serrano, S. M. , S. Begueria , and J. I. Lopez‐Moreno . 2010a. “A Multiscalar Drought Index Sensitive to Global Warming: The Standardized Precipitation Evapotranspiration Index.” Journal of Climate 23, no. 7: 1696–1718. 10.1175/2009jcli2909.1.

[ece370999-bib-0036] Vicente‐Serrano, S. M. , S. Beguería , J. I. Lopez‐Moreno , M. Angulo , and A. El Kenawy . 2010b. “A New Global 0.5° Gridded Dataset (1901–2006) of a Multiscalar Drought Index: Comparison With Current Drought Index Datasets Based on the Palmer Drought Severity Index.” Journal of Hydrometeorology 11, no. 4: 1033–1043. 10.1175/2010jhm1224.1.

[ece370999-bib-0037] Wilcox, K. R. , A. T. Tredennick , S. E. Koerner , et al. 2017. “Asynchrony Among Local Communities Stabilises Ecosystem Function of Metacommunities.” Ecology Letters 20, no. 12: 1534–1545. 10.1111/ele.12861. 29067791 PMC6849522

[ece370999-bib-0038] Xu, Z. , H. Sun , T. Zhang , H. Xu , D. Wu , and J. Gao . 2024. “The High Spatial Resolution Drought Response Index (HiDRI): An Integrated Framework for Monitoring Vegetation Drought With Remote Sensing, Deep Learning, and Spatiotemporal Fusion.” Remote Sensing of Environment 312: 114324. 10.1016/j.rse.2024.114324.

[ece370999-bib-0039] Yachi, S. , and M. Loreau . 1999. “Biodiversity and Ecosystem Productivity in a Fluctuating Environment: The Insurance Hypothesis.” Proceedings of the National Academy of Sciences of the United States of America 96, no. 4: 1463–1468. 10.1073/pnas.96.4.1463. 9990046 PMC15485

[ece370999-bib-0040] Zhang, Q. , D. Kong , V. P. Singh , and P. Shi . 2017. “Response of Vegetation to Different Time‐Scales Drought Across China: Spatiotemporal Patterns, Causes and Implications.” Global and Planetary Change 152: 1–11. 10.1016/j.gloplacha.2017.02.008.

